# Impact of Procedural Steps and Cryopreservation Agents in the Cryopreservation of Chlorophyte Microalgae

**DOI:** 10.1371/journal.pone.0078668

**Published:** 2013-11-11

**Authors:** Tony V. L. Bui, Ian L. Ross, Gisela Jakob, Ben Hankamer

**Affiliations:** Institute for Molecular Biosciences, The University of Queensland, Brisbane, Queensland, Australia; University of Sydney, Australia

## Abstract

The maintenance of traditional microalgae collections based on liquid and solid media is labour intensive, costly and subject to contamination and genetic drift. Cryopreservation is therefore the method of choice for the maintenance of microalgae culture collections, but success is limited for many species. Although the mechanisms underlying cryopreservation are understood in general, many technical variations are present in the literature and the impact of these are not always elaborated. This study describes two-step cryopreservation processes in which 3 microalgae strains representing different cell sizes were subjected to various experimental approaches to cryopreservation, the aim being to investigate mechanistic factors affecting cell viability. Sucrose and dimethyl sulfoxide (DMSO) were used as cryoprotectants. They were found to have a synergistic effect in the recovery of cryopreserved samples of many algal strains, with 6.5% being the optimum DMSO concentration. The effect of sucrose was shown to be due to improved cell survival and recovery after thawing by comparing the effect of sucrose on cell viability before or after cryopreservation. Additional factors with a beneficial effect on recovery were the elimination of centrifugation steps (minimizing cell damage), the reduction of cell concentration (which is proposed to reduce the generation of toxic cell wall components) and the use of low light levels during the recovery phase (proposed to reduce photooxidative damage). The use of the best conditions for each of these variables yielded an improved protocol which allowed the recovery and subsequent improved culture viability of a further 16 randomly chosen microalgae strains. These isolates included species from *Chlorellaceae, Palmellaceae, Tetrasporaceae, Palmellopsis, Scenedesmaceae* and *Chlamydomonadaceae* that differed greatly in cell diameter (3–50 µm), a variable that can affect cryopreservation success. The collective improvement of each of these parameters yielded a cryopreservation protocol that can be applied to a broad range of microalgae.

## Introduction

Microalgal biotechnologies are rapidly being developed for commercial exploitation of a range of products including health supplements, animal feeds, biofuels and chemical feed stocks [1]. It is estimated that approximately 350,000 microalgae species [2] may exist in a diverse range of habitats, ranging from fresh to saline water sources and from arctic conditions to thermal springs [3]. This biodiversity provides an excellent basis for future microalgae breeding programs which depend on extensive culture collections.

The maintenance of liquid or semisolid algae collections based on agar, though well-established, is labour intensive, costly and subject to contamination and genetic change [4]. Cryopreservation provides the most robust alternative approach for storage but has yielded varying degrees of success for microalgae [5], [6]. There is consequently a need for improved cryopreservation techniques. The aim of this paper is to improve our understanding of the key steps in the cryopreservation process and to optimize each of these.

Cryopreservation techniques are based on two general concepts; dehydration of cells using osmolytes or compatible solutes including sugars or polyols, and the prevention of ice crystal formation through the use of cell penetrating agents such as dimethyl sulfoxide and methanol [4], [6].

Sugars such as sucrose typically do not cross cell membranes unless active transporters are present in the outer membrane [7]. As the extracellular concentration is increased, an efflux of intracellular water is therefore generally thought to occur via osmosis which induces cell dehydration. This in turn reduces the unbound intracellular water available for ice crystal formation upon freezing [8]. Generally low molecular weight compounds are used [5], [9] but there have also been reports that certain high molecular weight unpenetrative cryoprotectants (i.e. dextran, polyvinylpyrrolidone) have been shown to have a cryoprotective effect on cell viability [5], although protection is reported to be limited.

Ice crystal formation can also be reduced by the addition of sulfoxides or alcohols such as dimethyl sulfoxide (DMSO) and methanol [5]. Both DMSO and methanol freely permeate cell membranes due to their low hydrophilicity and molecular weight and are therefore thought to disrupt ice crystal nucleation and formation by forming hydrogen bonds with water [10], [11], [12].

As both cellular dehydration and hydrogen bond disruption [5] can contribute to the reduction of ice crystal formation, the combined use of sugars and sulfoxides/alcohols in cryopreservation protocols can reportedly improve the success rate of cryopreservation [13].

Despite the fact that dehydration and the inhibition of ice crystal formation represent two distinct mechanisms by which cell damage can be reduced, there are few reported methods that use a combined strategy. In mammalian systems, Kuji et al. [14] and Shier and Olsen [15] are among the few studies in which sucrose and DMSO, used in combination, showed improved cell viability. For microalgae, mixtures of proline, ethylene glycol and DMSO showed better results than single agents for four species [15] while DMSO with sorbitol (or to a lesser extent sucrose) proved satisfactory for cryopreservation of thallus from the macroalga *Porphyra* [9], [16].

In addition to the strategies used to simultaneously increase dehydration and reduce ice crystal formation, a range of freezing protocols have been reported [4]. These include rapid single-step freezing protocols (e.g. plunging into liquid nitrogen to minimize ice crystal formation), two-step freezing (dehydration and cold acclimation, followed by rapid freezing to minimise ice crystal formation) [6] and controlled rate freezing processes (to minimize ice crystal formation and cell damage with or without cryoprotectants) [4]. Encapsulation techniques utilising sodium alginate have also been used, ostensibly to stabilise the cell membrane [17].

Of this wide range of protocols, the two-step cryopreservation process has been reported to be among the most robust and reliable techniques yielding high recovery levels [4], [18]. Osorio et al. [19] reported a recovery rate of 82% (of 100 algae strains cryopreserved) during their initial cryopreservation screen on the Coimbra collection. An added advantage of the two-step process is that it is easy and fast to perform [20] compared to other methods such as encapsulation and dehydration techniques [21] and does not require complex or expensive equipment.

**Figure 1:**
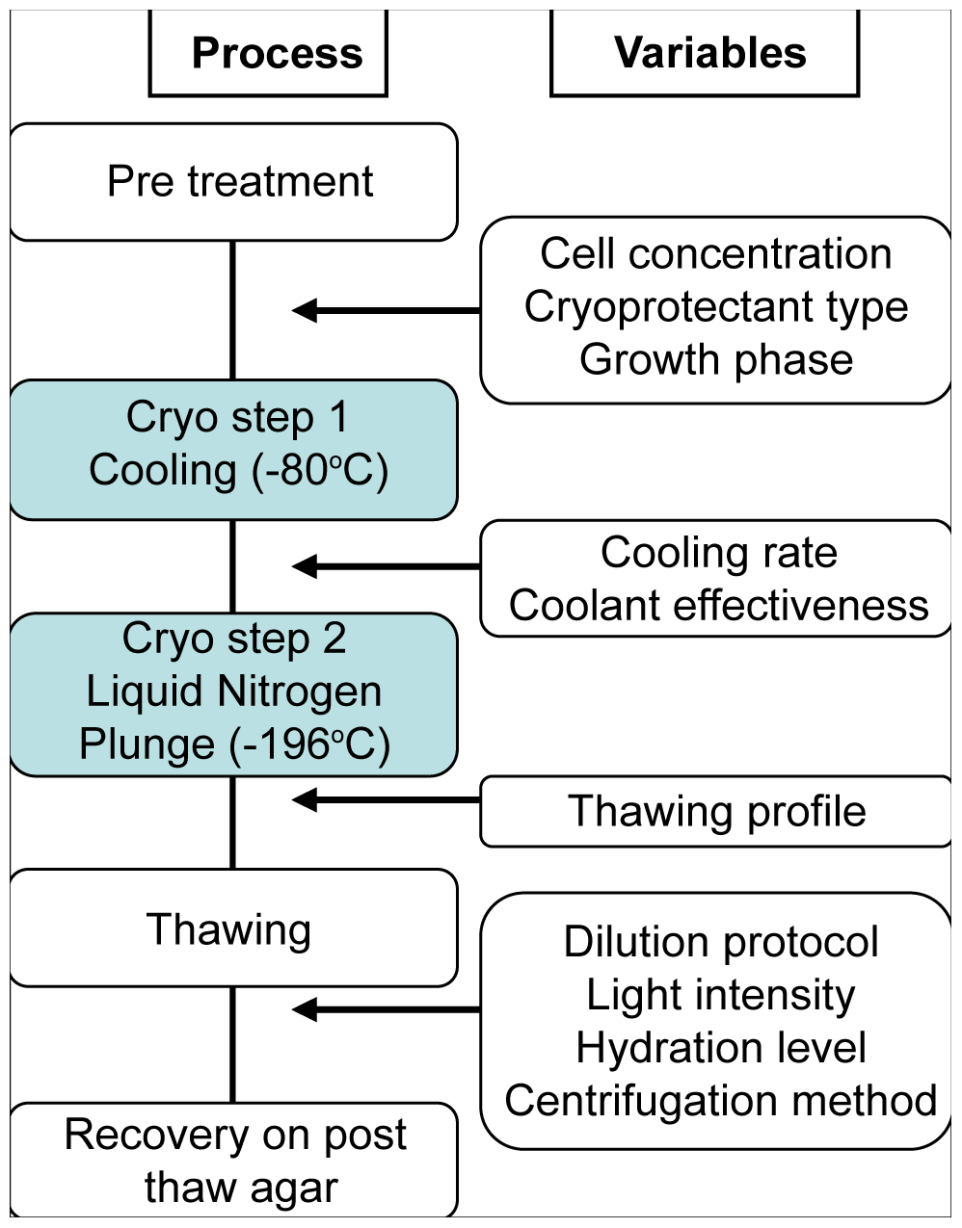
Overview of the cryopreservation process. Pretreatment Stage: Pretreatment of samples with cryoprotectants, cell concentration and growth stage; Cryo step 1: Samples freezing at a steady cooling rate. Cryo step 2: Liquid nitrogen plunge for long term storage. Recovery: Rapid thaw in room temperature water bath, cell dilution and agar plating for recovery.

The first stage of the two-step process is designed to dehydrate cells in a controlled manner. During this process, extracellular ice forms first and has the effect of concentrating solutes in the extracellular medium, raising the osmolarity of the extracellular solution, driving osmotic water loss from the cell and consequently cellular dehydration [4]. This dehydration process also increases the relative concentration of cell permeable cryoprotectants such as DMSO [8]. Once frozen, the second step of cryopreservation (See Figure 1 Cryo step 2) consists of plunging the cells into liquid nitrogen (−196°C) in preparation for long term storage. This paper describes an empirical investigation of key steps in this procedure and their relative importance in the success of cryopreservation.

## Methods

### Routine Microalgal Culture and Maintenance

Microalgae were routinely cultured using tris-acetate-phosphate (TAP) medium [22], [23] or TAP lacking acetate (tris-phosphate; TP medium) and stored at light intensities below 20 µE m^−2^ s^−1^. Photosynthetically Active Radiation (PAR) was measured using a handheld meter (Biospherical Instruments Inc). Microalgae cultures during late log or early stationary phase were used for experimentation as previously noted [19], [24]. A growth curve was established for each strain and the cell density at the onset of stationary phase was noted and used subsequently to signal the attainment of this stage of growth (assuming the employment of the same culture conditions). The final cryopreservation protocol (Fig. 1) is provided below, with variations noted in the results section.

### Isolation of Strains for Microalgae Culture Collection

Microalgae were isolated from local water bodies at a range of locations around Australia, but mostly from the Brisbane region. No specific permits were required for the algae collections conducted in these public waterways for research purposes. Three microalgae isolates were derived from private property for which permission was granted by the owners. Endangered and/or protected species were not involved. Full details of the collections will be described elsewhere (in preparation).

**Figure 2:**
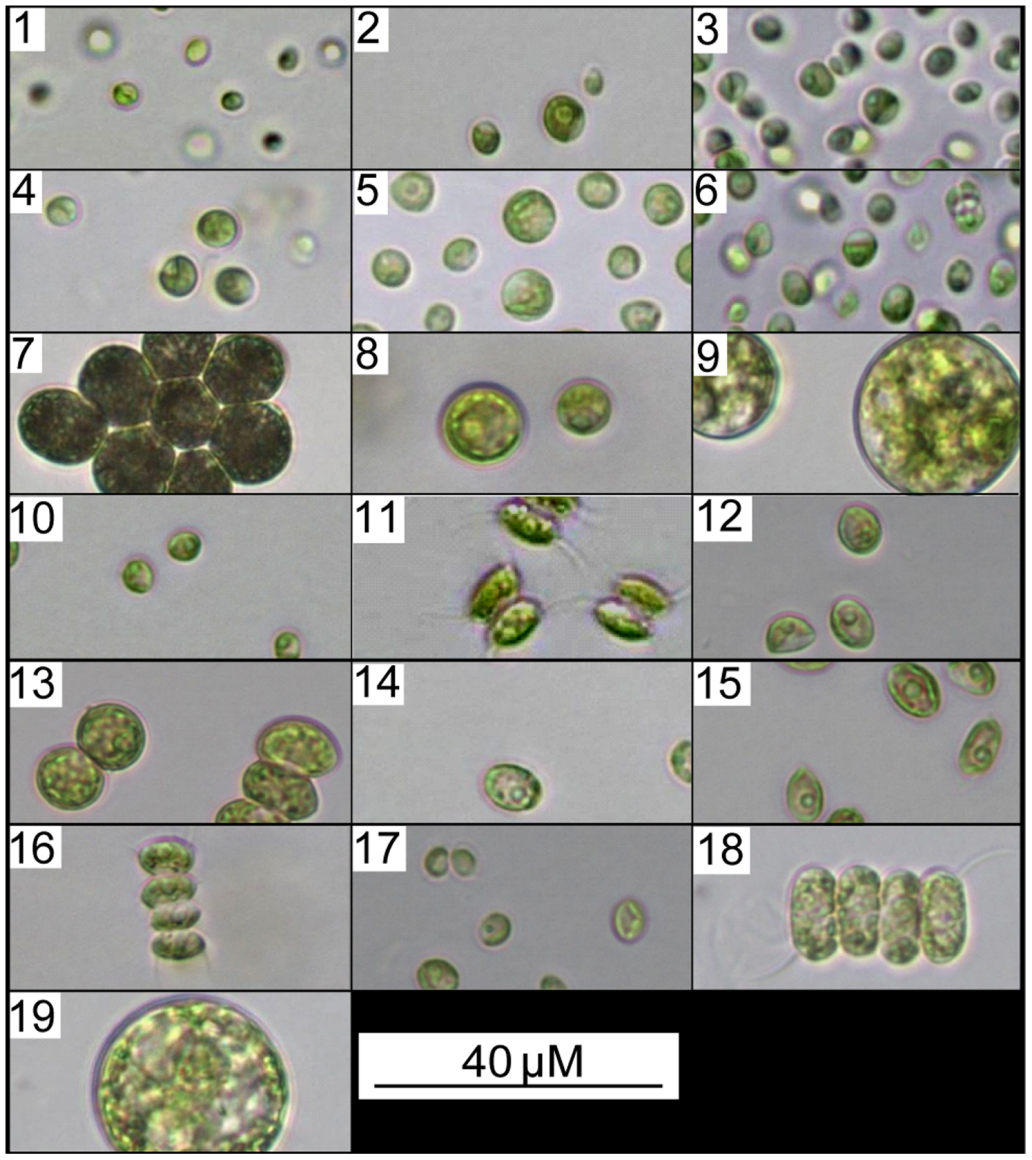
Representative algal strains showing successful recovery from cryopreservation. These chlorophyte strains were isolated from the local environment as axenic cultures. Strains 1–6, 8–9 and 19 *Palmellopsis* sp; 7 *Sphaerocystis* sp; 10, 17 *Tetraspora* sp; 11, 13, 16, 18 *Scenedesmus* sp; 12, 14–15 *Chlamydomonas* sp. All were able to be cryopreserved and showed improvements in viability after several optimisations of cryopreservation variables despite a range of cell sizes. Scale bar represents 40 µm.

Briefly, strains were isolated by dilution plating into 96 well plates, by plating onto agar containing either TP medium or autoclaved water from the sample source, or by growth in liquid medium (TP or water source). Dilution plating from enriched cultures or micromanipulation were used to isolate single algal species from colonies on plates, and single-cell fluorescence activated cell sorting (FACS) was used to isolate axenic cultures either by collecting into liquid medium or onto agar in 96 well plates. DNA analysis showed no bacterial ribosomal DNA sequences present in the axenic cultures. Once isolated, strains were maintained in suitable liquid or semisolid media. Strains used during this study are from the families *Chlorellaceae, Palmellaceae, Tetrasporaceae, Palmellopsis, Scenedesmaceae* and *Chlamydomonadaceae*. Taxonomy was derived by morphological comparison to a taxonomic guide [25]. The taxonomic identity of each strain number is provided in the Figure 2 legend.

### Initial Protocol

The initial protocol used as a starting point for method development was derived from the literature [4], [6], [8]. This involved the suspension of cells in 1.8 mL cryovials (Thermo Scientific Nunc) at undiluted culture concentrations typically in the range of 2 – 9×10^7^ cells mL^−1^. DMSO was added to the cultures to a final concentration of 10% (v/v). The cryovials were then inserted into a Nalgene Mr Frosty freezing apparatus, cooled in −80°C freezers overnight before being plunged in liquid nitrogen. The samples were then stored under these conditions for at least 24 hours. Cryovials were defrosted to room temperature, transferred to 1.5 mL microcentrifuge tubes and centrifuged at 1500 g for 8 minutes. The supernatant was discarded and resuspended with fresh TAP media. The sample was then serially diluted to a cell concentration of 2.5×10^4^ cells mL^−1^ and 200 µL was plated into TAP agar plates. Samples were left under light intensities from 30 – 150 µE m^−2^ s^−1^ constant illumination.

### Development of an Improved Protocol

Strains 3, 12 and 19 were used in this study to test the impact of particular steps of the protocol, as described below. Once a final protocol had been developed, a further 16 randomly selected strains varying in size (shown in Figure 2) as well as three type culture collection strains (*Chlorella vulgaris*, *Chlamydomonas reinhardtii* and *Scenedesmus dimorphus UTEX*) were trialled to compare the developed protocol to the initial protocol. Experiments were conducted in triplicates and post thaw viability was measured as colony forming units (CFU).

### Final Two-Step Cryopreservation: Pre-freeze

Microalgae were cultured in 100 mL flasks shaken at 100 rpm at an illumination of ∼90 µE m^−2^ s^−1^ in TAP medium. Cell concentrations were determined using a haemocytometer and the concentration adjusted to 5×10^6^ cells in 50 µL aliquots. Aliquots of the appropriate cell culture (containing 1×10^8^ cells ml^−1^) were then dispensed into cryovials (Nunc 1.8 mL).

Cryoprotectants including sucrose (final concentration 200 mM) and DMSO (final concentrations of 0.5–15%, Sigma Aldrich) were dispensed into separate 1.5 mL microcentrifuge tubes and gently mixed with TAP medium to yield a total volume of 950 µL. The cryoprotectant mix was slowly and gently pipetted into the cryovials containing the cells, so that during mixing, the effective cryoprotectant concentration was always less than the final desired concentration.

Cryovials of replicate algal samples were then inserted into a Nalgene ‘Mr Frosty’ freezing container (at room temperature), which had been filled with the recommended level (250 mL) of isopropanol. The Mr Frosty container was then placed into a −80°C freezer (Fig. 1). Under these conditions the system is designed to cool at approximately −1°C min^−1^. The samples were allowed to cool for at least 2 h before any transfers were made to liquid nitrogen storage. Samples were left in liquid nitrogen for at least one day prior to thawing for viability tests.

### Final Two-Step Cryopreservation: Thawing & Plating of Cells

Falcon tubes (50 mL Becton Dickinson) containing 49 mL of TAP media were prepared so that the addition of 1 mL frozen cells yielded a final volume of 50 mL. To prepare the samples for transfer to a laminar air flow cabinet, cryovials were kept under dry ice to maintain their temperature near −78°C after removal from liquid nitrogen storage. The keep period was under 10 minutes. The samples were then thawed for 2 min afloat in a water-filled beaker (∼500 mL, 25°C), 4 cryovials at a time. The thawed cell cultures were then gently poured into the sterile Falcon tubes containing 49 mL of growth media at room temperature and left without mixing for 1 h to avoid damage to the fragile cells. After 1 h the samples were gently mixed by carefully inverting the Falcon tubes 5 times. During this time, samples were subjected to light intensities ∼55 µE m^−2^ s^−1^ in the laminar flow hood.

In preparation for plating, TAP agar was poured fresh, 20 minutes prior to use (to minimize dehydration of the agar surface) and allowed to cool. Agar plates were then divided in two, one half being plated with a volume equivalent to 5×10^3^ cells from the original culture, and the other with 1×10^4^ cells. The surface of the agar plates was allowed to dry briefly, with care to prevent excessive drying. Each plate was then sealed against dehydration with Parafilm or plastic wrap (Fig. 1).

Cells inoculated in liquid culture were only used as a basis to determine whether cryopreserved cells could be recovered at all, in the case that they did not grow on solid media (i.e. survival <∼0.1%). No CFU assays were conducted on liquid cultures.

### Final Two-Step Cryopreservation: Post-Plating

Plates were stored at a range of light intensities (0–425 µE m^−2^ s^−1^ constant illumination). Cell colonies typically grew to visually detectable levels after 5–28 days. At this point, cell colonies were counted and percentage viabilities calculated, comparing observed CFU to the theoretically plated number of cells (based on the initial CFU count prior to cryopreservation).

### Investigation of Protocol Steps

#### Cell density and centrifugation studies

Microalgae cultures from strains 3 (*Palmellopsis* sp), 12 (*Chlamydomonas* sp) and 19 (*Palmellopsis* sp) were subjected to cryopreservation (final method above) at four concentrations in duplicate experiments. Cultures were diluted to 1, 5, 10 and 68×10^6^ cells mL^−1^ prior to cryopreservation and post thaw viability was measured as CFUs.

Strain 12 was also subjected to centrifugation studies involving four separate conditions conducted in three independent experiments. The conditions involved a centrifugation (1.5×10^3^ *g*) versus non-centrifugation step during the cryopreservation process. Each of the conditions also employed a diluted (1×10^6^ cells mL^−1^) vs. undiluted (5×10^7^ cells mL^−1^) culture concentration. Post thaw viability was measured as CFUs.

#### Cryoprotectant combinations and DMSO concentrations

Four conditions were tested; control (growth media only), sucrose only (final concentration 200 mM), 10% (v/v) DMSO only and 10% (v/v) DMSO and sucrose (final concentration 200 mM). The study was conducted in biological triplicates and post thaw viability was measured as CFUs. All 19 strains were used in this study (Fig. 2).

Test strains 3, 12 and 19 were subjected to various concentrations of DMSO during cryopreservation. The concentrations were 0, 0.5, 3, 4, 5, 6, 7, 12, 15% (v/v) during the cryopreservation process (refer to method above). This study was conducted in three independent experiments and post thaw viability was measured as CFUs.

#### Sucrose studies

Strains 3, 12 and 19 were subjected to the cryopreservation process with the addition of sucrose into the cryoprotectant mix before (described above) or after cryopreservation. The sucrose concentrations in both conditions were 5 mM at the point of diluting samples into 50 mL Falcon tubes. The study was conducted in three independent experiments and post thaw viability was measured as CFUs.

#### Light intensity

Microalgae strains 3, 12 and 19 were cryopreserved (detailed above) and subjected to varying light radiances during the post thaw step at 0, 3, 8, 22, 56, 71, 169 and 425 µE m^−2^ s^−1^. Experiments were conducted in triplicates and post thaw viability was measured as CFUs.

## Results and Discussion

This paper describes the empirical investigation of key steps in cryopreservation (Fig. 1) and their integration into a process with enhanced cryopreservation efficiency. Each of the key variables was initially tested and optimized using 3 microalgae test strains before validating the combined process using 19 strains. These 19 microalgae species differed in size (3–50 µm) and morphology (Fig. 2) and were selected at random from a collection of locally isolated strains. Ultimately, the aim of the optimised protocol was to increase the viability of each strain.

### Cell Density

Figure 1 shows the typical design of a two-step freezing protocol and the factors that can affect its success. Optimization began with cell concentration, as Piasecki et al. [24] reported that high cell concentrations reduce cryopreservation efficiency, apparently due to the presence of an injurious substance (MW< 1500 Da), which was enzymatically released from the cell wall upon cell death [24]. This conclusion was based on heat denaturing algae cells (to 95°C) before and after the substance was released which proved that heat prevented the (presumably enzymatic) release of the substance. It was further supported by using cell wall deficient mutants, which showed concentration-independent viability [24].

**Figure 3:**
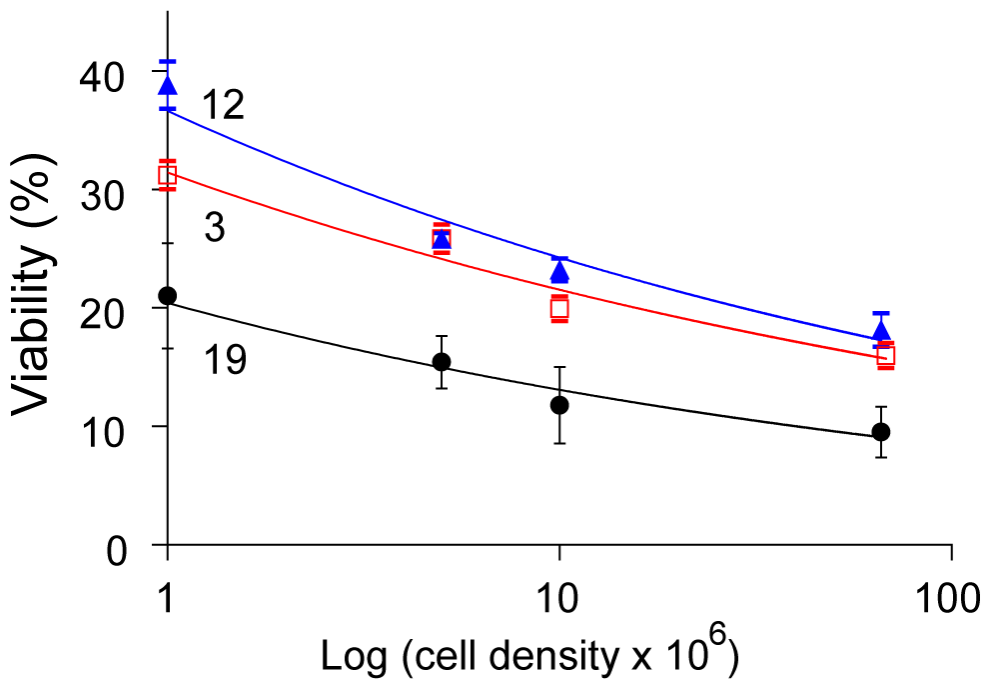
The effect of cell concentration on cryopreservation efficiency. Cell viability of strains 3 (red square), 12 (blue triangle) and 19 (black circle) expressed as % CFU / theoretically plated cells. Results of duplicate experiments are shown for 1, 5, 10 and 68 million cell mL^−1^. Error is represented as the standard error of the mean and lines of best fit are spline fits.

Consequently, initial experiments tested the effect of cell concentration (Figure 3) on cryopreservation efficiency in the presence of 200 mM sucrose and 10% DMSO (v/v). These sucrose and DMSO concentrations were used as the starting formulation of the freezing buffer based on previous reports [4], [5]. Figure 3 shows the effect of cell concentration observed for three representative strains. These were selected to represent three different sizes as shown in Figure 2, namely, strain 3 (3–5 µm diameter), strain 12 (12–15 µm diameter) and strain 19 (25–35 µm diameter). Figure 3 shows that upon increasing the cell concentration from 1×10^6^ to 10×10^6^ cell mL^−1^ the viability dropped from ∼37% to ∼24% but did not greatly decline further at higher cell densities. In all cases, higher viabilities were obtained at lower cell concentrations, consistent with the report of Piasecki et al. [24]. The fact that the curve flattens at higher cell concentrations suggests that postulated toxic compounds released from dying cells might not be lethal to all cells in the concentration range tested. As the highest recovery was seen at 1×10^6^ cell mL^−1^ this concentration was used as the default concentration for subsequent experiments. We cannot however exclude the possibility that further dilution could further improve recovery rates, as viability assays were not conducted for cell concentrations below 1×10^6^ cells mL^−1^. Although improved viability at lower cell concentrations appears advantageous, lower cell concentrations mean a substantial reduction in the absolute numbers of colonies, even with an improved survival, and so is of limited interest.

### Centrifugation of Thawed Cells

Centrifugation can impose shear forces that can be damaging to algal cells, especially post-thaw samples when they are fragile [26]. For this reason the effect of centrifugation, which is also used to prepare cells for pre-cooling, was tested.

**Figure 4:**
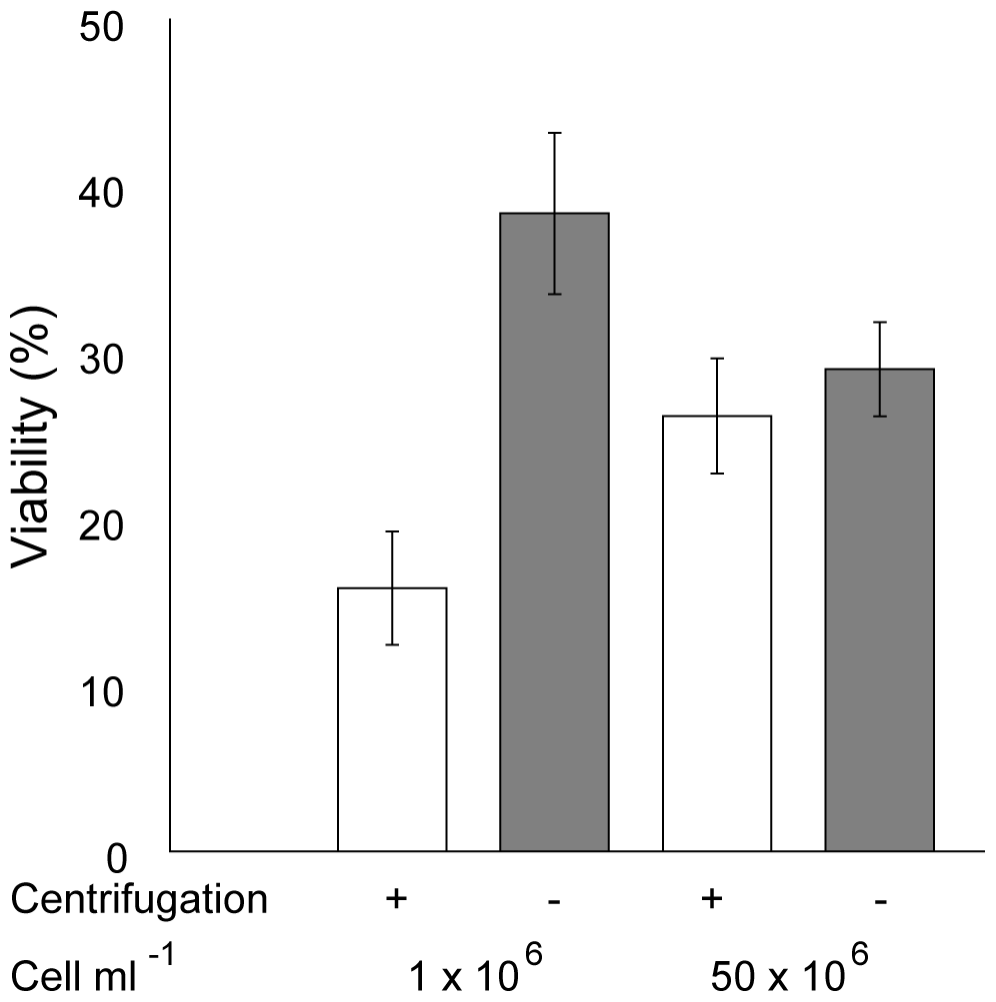
The effect of centrifugation (600×*g* for 10 min) on post thaw strain 12 (*Chlamydomonas* sp.) at 1×10^6^ and 50×10^6^ cells mL^−1^. Cell viability was calculated based on CFU / theoretically plated cells. White bars represent samples that were centrifuged whilst grey bars represent uncentrifuged samples. Error is represented as the standard deviation of three individual experiments.

Figure 4 demonstrates that eliminating the centrifugation step post thaw increased cell viability by ∼22% (of total cryopreserved cells) at 1×10^6^ cells mL^−1^ with a nonsignificant rise of ∼4% at 50×10^6^ cells mL^−1^. The observation that improved recovery was obtained at lower cell densities, is consistent with Figure 3. It cannot be excluded that the larger drop in viability observed for the 1×10^6^ cells mL^−1^ may in part be due to handling processes and particularly the adhesion of cells to the pipette and the micro centrifuge tubes, which will have a greater effect at lower concentrations. However, as centrifugation was not essential to the post thaw recovery steps, and as the observed effects were generally negative, it was eliminated from the protocol.

### Cryoprotectant Combinations

In preliminary screens, several cryoprotectants were examined, including methanol which is used as a standard method for *Chlamydomonas reinhardtii,* although it has also been reported to induce light and temperature sensitivity [27], [28]. Others included glycerol, sucrose, trehalose and DMSO (data not shown). Of the cryoprotectants initially tested, sucrose and DMSO proved to be the most effective for the strains used here, and were therefore selected for further analysis.

**Figure 5:**
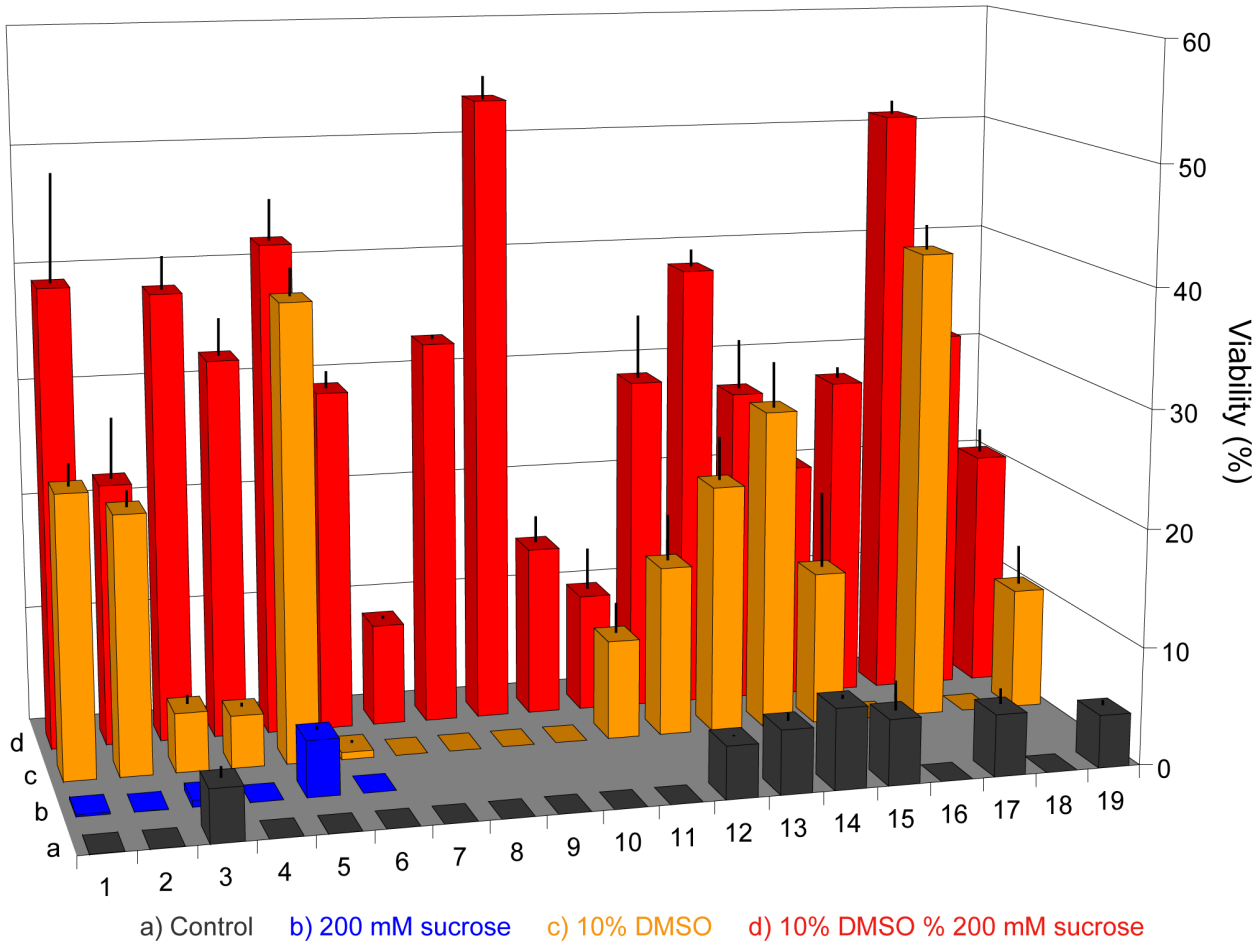
Effect of sucrose, DMSO and sucrose/DMSO mixtures on cryopreservation recovery. Row 1 (black): Control lacking sucrose and DMSO. Row 2 (blue): 200 mM sucrose. Row 3 (orange): 10% DMSO. Row 4 (red), 200 mM sucrose and 10% DMSO. Numbers represent different algae isolates. The data values are an average of triplicates and error bars represent the standard deviation.

In the control sample (Figure 5, no sucrose or DMSO), only 7 out of the 19 strains tested were recovered (37% success) and then only with low viability (i.e. <10% of cells plated were recovered as individual colonies). The effect of sucrose as a sole cryoprotectant was only tested on strains 1–6 (Figure 5 blue) and yielded low viability levels for 3 of 6 strains. DMSO had a significantly greater cryoprotective effect (68%: 13 of 19 strains recovered). Successfully recovered strains also exhibited improved viability (i.e. 50% of cells plated were recovered as individual colonies).

The combined use of sucrose and DMSO yielded a further marked improvement both in terms of the number of strains recovered (19 out of 19; 100% success) as well as for the cell viability (∼55% of cells plated were recovered as individual colonies) under the conditions tested. This result initially appeared consistent with our working hypothesis that the addition of sucrose dehydrates the cells and simultaneously increases the intracellular concentration of DMSO which is able to freely permeate into the cells thereby increasing its effectiveness [29]. Three differently sized strains (3, 12 and 19) were then selected to trial specific interventions in a more detailed fashion. These included the effects of DMSO concentration and light intensity.

### Effect of DMSO

**Figure 6:**
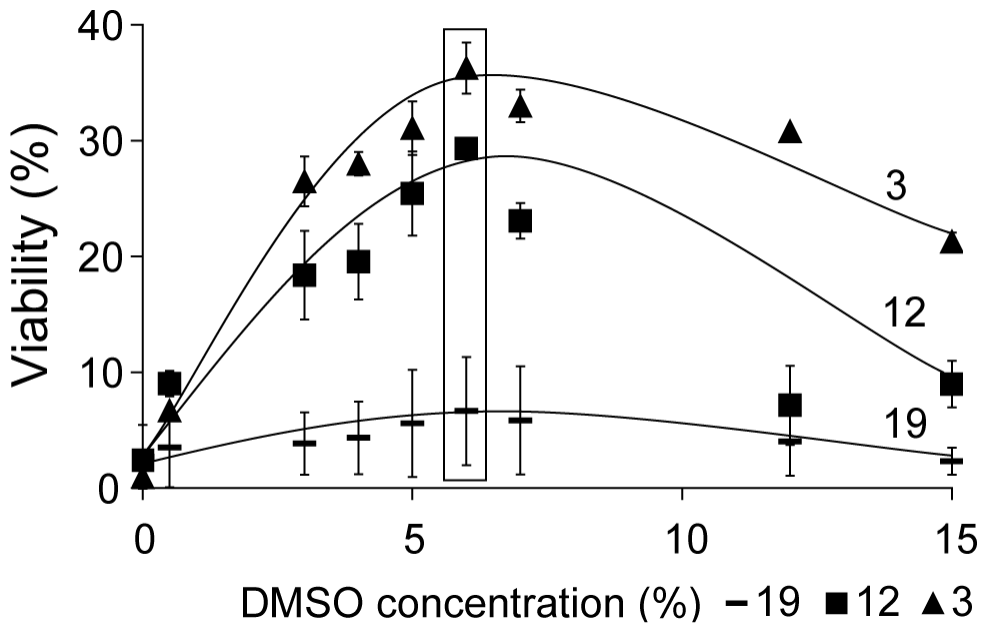
Effect of DMSO concentration. Strains 3 (triangle), 12 (square) and 19 (rectangle) all yielded optimal recoveries in terms of % cell viability at 6.5% DMSO. Each data point was is the average of three independent experiments. Error bars indicate the standard deviation and lines of best fit are spline fits.

Although the combination of sucrose and DMSO (Figure 5) yielded higher cell viabilities for all strains tested, some strains still demonstrated low viabilities compared to others (i.e. strain 9 and 17 with viability over 50% vs. strain 7 and 11 with viability under 10%). As the morphology of these strains was variable (Figure 2), morphology or size was not relied upon as an explanation. Instead it was assumed that DMSO, while an effective cryoprotectant, could also be harmful to some microalgal strains. A range of DMSO concentrations were tested with the three selected strains, to identify if there was an optimum range (Figure 6).

In all three cases, DMSO concentrations between 6–7% provided the best cryoprotection though concentrations from 5–10% were acceptable. Reduced cell viabilities observed at lower DMSO concentrations are thought to be due to reduced efficacy in terms of cryopreservation (Figure 6). Reduced cell viability at DMSO concentrations above 6.5% may indicate harmful effects, for example on the integrity of cell membranes. Based on these results a 6.5% DMSO concentration was defined as optimal for these three strains. Although strictly, this does not permit extrapolation to other strains, the similar curves and the proposed mechanisms suggest the findings are applicable to a wider range of microalgae.

### Effect of Sucrose

**Figure 7:**
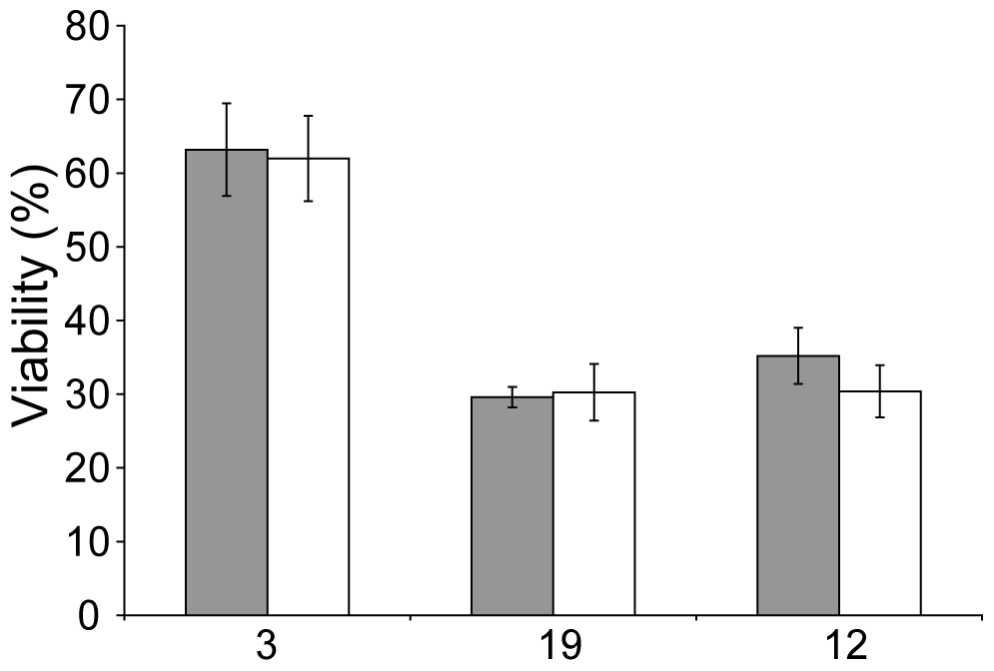
Effect of sucrose as a cryoprotectant. No significant differences in % viability were observed when strains 3, 12 and 19 underwent cryopreservation with sucrose added before (grey) or after the process (white). Error bars indicate the standard deviation of three independent experiments which were calculated from CFUs.

Sugars which are unable to diffuse across the cell membrane are often used as cryoprotectants. We tested sucrose, glucose, fructose and trehalose and found that only sucrose and glucose contributed to improved cryoprotection (data not shown) which suggested that they were not acting as simple osmolytes. We also noticed that the colony size was much larger on glucose and sucrose. Since these “cryoprotectants” are used in high concentration and some inevitably carries over into the recovery medium, it was possible that they were contributing to heterotrophic growth during the recovery phase. We therefore compared the recovery of cells when sucrose was added either as a cryoprotectant before freezing, with those where a small amount of sucrose was added only after thawing, in the same concentration as the carryover from thawing and washing. We found no difference in recovery (Figure 7), signifying that the effectiveness of sucrose most likely lies in the post-thaw recovery phase, and not as an osmolyte during the freezing step. This also suggests that the effectiveness of sucrose or other sugars will sometimes depend on the ability of microalgae to utilise them as heterotrophic growth substrates. In this particular instance, the lack of effectiveness of fructose suggests that only the glucose moiety of sucrose is being utilised as a growth substrate.

### Effect of Light Intensity

Light is required for photosynthesis, but at high levels results in photoinhibition, in large part due to the formation of reactive oxygen species [30]. Immediately after thawing, photosynthetic membranes may be damaged, more fragile and more susceptible to light-mediated damage. Consequently, relatively low light levels (30–150 µE m^−2^ s^−1^) were initially used on freshly plated cells with the aim of minimizing photodamage during the post thaw recovery phase. To more systematically evaluate the effect of light intensity on cell recovery, thawed cells were plated on TAP agar plates and illuminated at light levels ranging from 0–425 µE m^−2^ s^−1^ for approximately 2 weeks during which colonies were allowed to grow from the plated cells. As all three test strains (3, 12 and 19) were photoheterotrophic, the effect of complete darkness could also be examined by growth on TAP agar.

**Figure 8:**
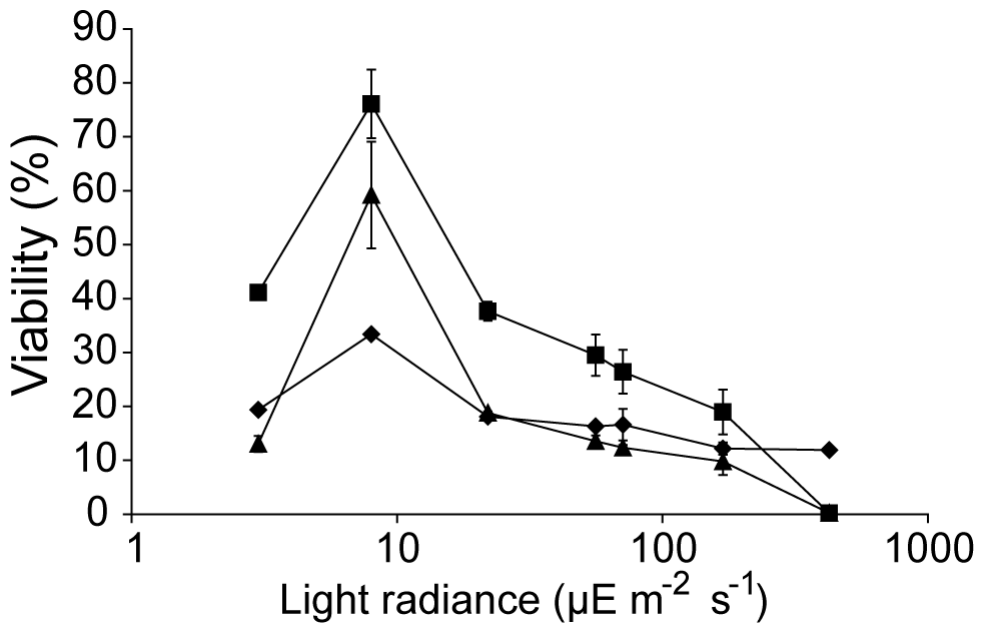
Cell recovery as a function of light intensity. Microalgae strains 3 (diamond), 12 (square) and 19 (triangle) were all most effectively recovered at 8 µE m^−2^ s^−1^ illumination. The viability was calculated from CFU per total number of plated cells. Error bars indicate the standard deviation of three independent experiments.

Microalgae cultures can typically be well maintained in the 40–60 µE m^−2^ s^−1^ illumination range, and up to ∼200 µE m^−2^ s^−1^ do not suffer photoinhibition. However these experiments indicated that during the recovery phase much lower light intensities (Figure 8; ∼8 µE m^−2^ s^−1^) were found to be optimal for growth. Figure 8 indicates that light is beneficial for recovery even in the presence of the acetate carbon source in TAP, but that at above ∼8 µE m^−2^ s^−1^ the cells in the recovery phase are very sensitive to photodamage.

All three test strains gave the highest rate of recovery at ∼8 µE m^−2^ s^−1^ illumination and this value was therefore used in the final improved cryopreservation protocol. Presumably this represents a compromise between the beneficial effect of light (particularly for ATP production) on cell viability and the harmful effect of light-mediated damage on photosynthetic membranes where integrity and repair mechanisms may be compromised by freeze-thaw damage.

### Protocol Comparisons

**Figure 9:**
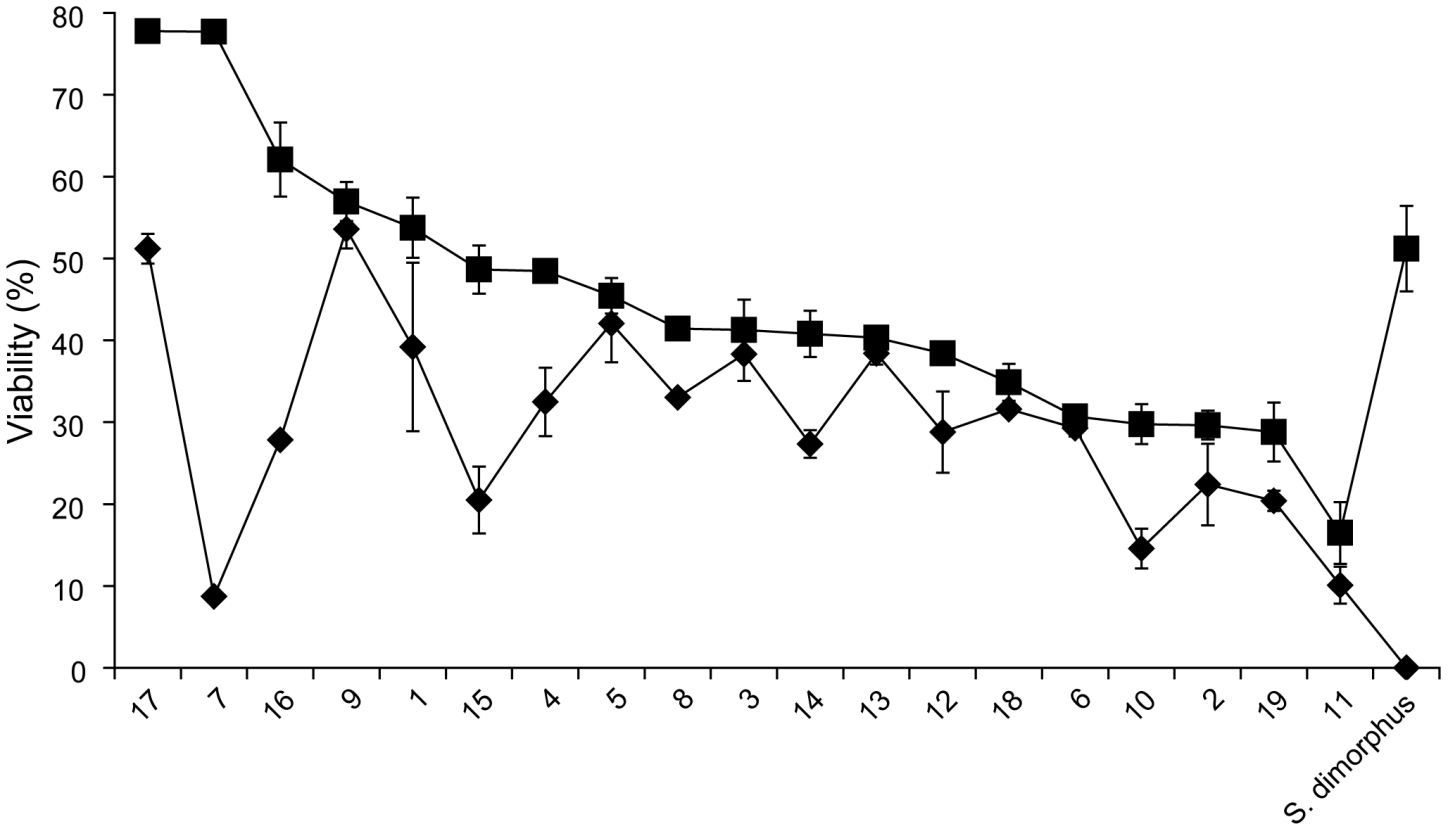
Cell recovery using initial and improved protocols. Strains are ordered from highest to lowest recovery rates using the improved protocol (square), and compared to the recovery rates for the initial protocol (diamond). Triplicate analyses were conducted and error bars represent the standard deviation. The algae size tested ranged from 3–50 µm.

Having defined the optimal settings for cell density (Figure 3), centrifugation (Figure 4), sucrose, DMSO, combined DMSO and sucrose (Figure 5) as well as DMSO concentration (Figure 6) and post thaw light exposure levels (Figure 8), each optimized setting was integrated into an ‘improved protocol’. Figure 9 compares the percent viabilities obtained with the initial protocol (Fig. 9 diamond which corresponds to Fig. 5, red) and the improved protocol (Figure 9 square) for all of the strains shown in Figure 2. The protocol comparison experiment also included three reference strains, *Chlorella vulgaris*, *Chlamydomonas reinhardtii* and *Scenedesmus dimorphus*. The reference strains were additionally subjected to cryopreservation using species-specific protocols from the literature [15], [27], [31].

The improved protocol allowed the successful recovery of all 19 trial strains and in all cases with improved viability. Figure 9 also demonstrates that the method is useful for microalgae ranging in size from 3–50 µm in diameter. This size range covers a broad range of algae, although our microalgae collections still contain very large strains that have proved more difficult to cryopreserve. Micrographs of strain cultures that still had relatively low viability revealed no clear trends in in terms of size, morphology or taxonomy, for example strains 11 (10 µm; 16.5 %) and 2 (8 µm; 29.6 %). It proved however to yield a high success rate for a broad range of microalgae sizes without the need for strain-specific testing or optimisation. Of the reference strains, all were recoverable in liquid media (regardless of the protocol used), however S. *dimorphus* was the only strain recoverable on solid media. This observation is in line with Crutchfield et al. [27] as their cryopreservation attempts for C. *reinhardtii* yielded high viability in liquid media in contrast with solid media. It was believed that even though individual cells may survive the cryopreservation, they may not survive the harsher conditions presented by growth on solid media. It is encouraging that our improved protocol, albeit not as effective as species specific protocols, was still capable of cryopreserving broader ranges of microalgae with good viability figures.

The examination of key variables suggests that further improvements are possible. After the homogenous ice nucleation temperature (−40°C) has been reached, ice crystal formation becomes inevitable [32]. The basis of the 2-step method is the supposition that only intracellular ice, and not external ice, is harmful [6] though some studies indicate that the terminal transfer temperature is important [33]. Specific vitrification agents can be used (e.g. for nucellar embryos [34]) to completely inhibit ice formation, but have proven toxic to algae [35] and to date, attempts to vitrify algae have relied upon encapsulation in alginate followed by dehydration [17], [36]. A more comprehensive investigation of these variables is clearly of interest.

Most improvements noted here affected the post-thaw recovery phase, suggesting that while some cells will always be killed by freezing, many others can recover if treated correctly, resulting in successful recovery even for strains which are normally difficult to preserve.
